# Supplementary material for: Use of Prescribed Drugs to Treat Chronic Diseases during Pregnancy in Outpatient Care in Switzerland between 2014 and 2018: Descriptive Analysis of Swiss Health Care Claims Data
Source: Int J Environ Res Public Health. 2022 Jan 27;19(3):1456. doi: 10.3390/ijerph19031456 (PMC8835338; doi:10.3390/ijerph19031456)
Supplement: Supplementary file 1 [file ijerph-19-01456-s001.zip › ijerph-1521655-supplementary.pdf]

Table S1. Description of the study population (weighted numbers).

Year	N of deliveries in the cohort	Mean of maternal age at delivery in the cohort (sd)	Mean maternal age at delivery in Switzerland (BfS)	Percentage of cesarean section in the cohort	Percentage of cesarean section in Switzerland (BfS)
**2014**	9’560	32.09 (5.06)	31.7	34.6	33.7
**2015**	9’409	32.10 (5.20)	31.8	34.8	33.3
**2016**	9’163	32.03 (5.25)	31.8	34.1	33.2
**2017**	8’811	32.12 (5.33)	31.9	33.8	32.3
**2018**	8’300	32.09 (5.23)	32.0	33.4	32.1

Table S2. Exposure prevalence to different drug groups and active substances during pregnancy overall and within trimester of pregnancy and pre-pregnancy separately (weighted numbers).

ATC code	Drug substance	Pre pregnancy N ((10'000)	T1 N (/10'000)	T2 N (/10'000)	T3 N (/10'000)	T1-T3 N (/10'000)
	**At least one drug from any of the following drug groups**	**5'267 (1'164.0)**	**4'837 (1'068.9)**	**5'052 (1'116)**	**7'020 (1'551.3)**	**9'939 (2'196.4)**
**H03**	**Thyroid therapy**	**1'287 (284.4)**	**2'115 (467.4)**	**2'316 (511.8)**	**2'139 (472.7)**	**3'021 (667.6)**
H03A	Thyroid preparations	1'244 (274.9)	2'071 (457.7)	2'283 (504.5)	2'113 (467.0)	2'967 (655.7)
H03AA01	*Levothyroxine sodium*	*1'227 (271.2)*	*2'057 (454.6)*	*2'268 (501.2)*	*2'100 (464.1)*	*2'945 (650.8)*
H03AA02	*Liothyronine sodium*	*21 (4.6)*	*15 (3.3)*	*15 (3.3)*	*15 (3.3)*	*25 (5.5)*
H03B	Antithyroid preparations	48 (10.6)	46 (10.2)	34 (7.5)	26 (5.7)	58 (12.8)
H03BA02	*Propylthiouracil*	*22 (4.9)*	*33 (7.3)*	*16 (3.5)*	*11 (2.4)*	*38 (8.4)*
H03BB01	*Carbimazole*	*30 (6.6)*	*22 (4.9)*	*20 (4.4)*	*16 (3.5)*	*37 (8.2)*
**C02, C03, C07, C08, C09**	**Anti-hypertensive drugs**	**340 (75.1)**	**301 (66.5)**	**583 (128.8)**	**2'077 (459.0)**	**2'431 (537.2)**
C08	Calcium channel blockers	32 (7.1)	51 (11.3)	348 (76.9)	1'733 (383.0)	1879 (415.2)
C08CA05	*Nifedipine*	*18 (4.0)*	*36 (8.0)*	*338 (74.7)*	*1'714 (378.8)*	*1'850 (408.8)*
C08CA01	*Amlodipine*	*10 (2.2)*	*13 (2.9)*	*	*16 (3.5)*	*25 (5.5)*
C07	Beta blocking agents	197 (43.5)	209 (46.2)	215 (47.5)	317 (70.1)	474 (104.7)
C07AG01	*Labetalol*	*28 (6.2)*	*70 (15.5)*	*100 (22.1)*	*210 (46.4)*	*259 (57.2)*
C07AB02	*Metoprolol*	*67 (14.8)*	*79 (17.5)*	*70 (15.5)*	*68 (15)*	*127 (28.1)*
C09	RAA acting agents	70 (15.5)	27 (6.0)	12 (2.7)	*	42 (9.3)
C09CA06	*Candesartan*	*	*	*	*	*
C09AA03	*Lisinopril*	*11 (2.4)*	*	*	*	*

C03	Diuretics	35 (7.7)	13 (2.9)	* (1.5)	16 (3.5)	34 (7.5)
C03CA01	*Furosemide*	*10 (2.2)*	*	*	*11 (2.4)*	*17 (3.8)*
C03CA04	*Torasemide*	*10 (2.2)*	*	*	*	*14 (3.1)*
C02	Antihypertensives	*	*	*	*	*
C02AB01	*Methyldopa*	*	*	*	*	*
C02CA04	*Doxazosin*	*	*	*	*	*
**N06A, N05A, N05AN, N05BA, N05CD, N05CF**	**Psychotropic drugs**	1'912 (422.5)	1'117 (246.8)	790 (174.6)	771 (170.4)	1'716 (379.2)
**N06A**	**Antidepressants**	**1'151 (254.4)**	**793 (175.2)**	**563 (124.4)**	**497 (109.8)**	**1'127 (249.1)**
N06AB	Selective serotonin reuptake inhibitors	689 (1.5)	505 (1.1)	398 (88)	355 (78.5)	726 (1.6)
N06AB10	*Escitalopram*	*286 (63.2)*	*173(38.2)*	*126(27.8)*	*117(25.9)*	*255 (56.4)*
N06AB06	*Sertraline*	*163 (36.0)*	*130 (28.7)*	*136 (30.1)*	*134 (29.6)*	*219 (48.4)*
N06AX	Other antidepressants	474 (104.7)	294 (65.0)	169 (37.4)	141 (31.2)	423 (93.5)
N06AX25	*Hyperici herba*	*112 (24.8)*	*74 (16.4)*	*63(13.9)*	*60 (13.3)*	*160 (35.4)*
N06AX16, N06AX21	*Serotonin and noradrenaline reuptake inhibitors (duloxetine, venlafaxine)*	*151 (33.4)*	*110 (24.3)*	*58 (12.8)*	*43 (9.5)*	*124 (27.4)*
N06AX11, N06AX03	*Alpha antagonists (mirtazapine, mianserine)*	*71 (15.7)*	*46 (10.2)*	*24 (5.3)*	*17 (3.8)*	*65 (14.4)*
N06AA	Non selective monoamine reuptake inhibitors	70 (15.5)	45 (9.9)	17 (3.8)	17 (3.8)	60 (13.3)

N06AA06	*Trimipramine*	*39 (8.6)*	*23 (5.1)*	*	*	*28 (6.2)*
N06AA09	*Amitriptyline*	*19 (4.2)*	*14 (3.1)*	*11 (2.4)*	*12 (2.7)*	*23 (5.1)*
**N05A**	**Antipsychotics**	**227 (50.2)**	**130 (28.7)**	**85 (18.8)**	**92 (20.3)**	**171 (37.8)**
N05AH04	*Quetiapine*	*93 (20.6)*	*63 (13.9)*	*54 (11.9)*	*56 (12.4)*	*82 (18.1)*
N05AX12	*Aripiprazole*	*30 (6.6)*	*20 (4.4)*	*13 (2.9)*	*15 (3.3)*	*29 (6.4)*
N05AH03	*Olanzapine*	*22 (4.9)*	*14 (3.1)*	*	*11 (2.4)*	*21 (4.6)*
N05AX08	*Risperidone*	*14 (3.1)*	*	*	*	*11 (2.4)*
N05AD01	*Haloperidol*	*	*	*	*	*10 (2.2)*
N05AD08	*Droperidol*	*46 (10.2)*	*	*	*	*
N05AF03	*Chlorprothixene*	*10 (2.2)*	*	*	*	*
N05AN01	*Lithium*	*	*	*	*	*
**N05BA, N05CD, N05CF**	**Benzodiazepine derivatives / related drugs**	**898 (198.4)**	***410 (90.6)***	***234 (51.7)***	***259 (57.2)***	***733 (162.0)***
N05BA, N05CD	Benzodiazepine derivatives	765 (169.1)	317 (70.1)	204 (45.1)	211 (46.6/10'00)	602 (133)
N05BA06	*Lorazepam*	*331 (73.1)*	*158 (34.9)*	*102 (22.5)*	*122(27.0)*	*329 (72.7)*
N05CD08	*Midazolam*	*238 (52.6)*	*45 (9.9)*	*11 (2.4)*	*14 (3.1)*	*65 (14.4)*
N05CF	Benzodiazepine related drugs	188 (41.5)	116 (25.6)	40 (8.8)	61(13.5)	177 (39.1)
N05CF02	*Zolpidem*	*169 (37.3)*	*108 (23.9)*	*39 (8.6)*	*60 (13.3)*	*167 (36.9)*
N05CF01	*Zopiclone*	*21 (4.6)*	*	*	*	*11 (2.4)*
**A10**	**Anti-diabetics**	440 (97.2)	389 (86.2)	551 (121.8)	1'565 (345.8)	1'786 (394.7)
A10A	Insulins and analogues	124 (27.4)	189 (41.8)	495 (1.1)	1'522 (336.3)	1'582 (349.6)
A10AE	Long acting	56 (12.4)	100 (22.1)	310 (68.5)	939 (207.5)	1'011 (223.4)
A10AB	Fast acting	110 (24.3)	153 (33.8)	252 (55.7)	714 (157.8)	749 (165.5)
A10AC	Intermediate acting	*	24 (5.3)	115 (25.4)	435 (96.1)	469 (1.0)
A10B	Blood glucose lowering drugs	330 (72.9)	221 (48.8)	70 (15.5)	65 (14.4)	273 (60.3)

A10BA02	*Metformin*	*308 (68.1)*	*212 (46.8)*	*66 (14.6)*	*58 (12.8)*	*255 (56.4)*
A10BJ02	*Liraglutide*	*	*	*	*	*
A10BB01	*Glibenclamide*	*	*	*	*	*
**R03**	**Obstructive airway diseases**	893 (197.3)	694 (153.4)	773 (170.8)	739 (163.3)	1'637 (361.8)
R03AK	Adrenergics with glucocorticoids or other drugs (excl anticholinergics)	624 (137.9)	426 (94.1)	479 (105.9)	425 (93.9)	968 (213.9)
R03AK07	*Formoterol and budesonide*	*421 (93.0)*	*273 (60.3)*	*330 (72.9)*	*277 (61.2)*	*673 (148.7)*
R03AK06	*Salmeterol and fluticasone*	*175 (38.7)*	*132 (29.2)*	*139 (30.7)*	*131 (28.9)*	*275 (60.8)*
R03AC	Selective B2 adrenoreceptor agonists	317 (70.1)	296 (65.4)	327 (72.3)	279 (61.7)	717 (158.4
R03AC02	*Salbutamol*	*272 (60.1)*	*252 (55.7)*	*283 (62.5)*	*249 (55.0)*	*637 (140.8)*
R03AC13	*Formoterol*	*25 (5.5)*	*30 (6.6)*	*19 (4.2)*	*16 (3.5)*	*43 (9.5)*
R03BA	Inhaled Glucocorticoids	78 (17.2)	95 (21.0)	118 (26.1)	98 (21.7)	255 (56.4)
R03BA02	*Budesonide*	*30 (6.6)*	*50 (11.0)*	*65 (14.4)*	*52 (11.5)*	*149 (32.9)*
R03BA05	*Fluticasone*	*27 (6.0)*	*24 (5.3)*	*34 (7.5)*	*27 (6.0)*	*65 (14.4)*
R03CC	Selective beta-2 adrenoreceptor agonists	*	*	*	59 (13.0)	61 (13.5)
R03CC05	*Hexoprenaline*	*	*	*	*56 (12.4)*	*56 (12.4)*
R03CC02	*Salbutamol*	*	*	*	*	*
R03BB	Anticholinergics	21 (4.6)	19 (4.2)	16 (3.5)	18 (4.0)	45 (9.9)
R03BB01	*Ipratropium bromide*	*16 (3.5)*	*16 (3.5)*	*13 (2.9)*	*16 (3.5)*	*40 (8.8)*
R03BB04	*Tiotropium bromide*	*	*	*	*	*
R03AL	Adrenergics in combination with anticholinergics	17 (3.8)	12 (2.7)	13 (2.9)	20 (4.4)	40 (8.8)

R03AL02	*Salbutamol and ipratropium bromide*	*114 (25.2)*	*89 (19.7)*	*74 (16.4)*	*124 (27.4)*	*259 (57.2)*
R03DC (R03DC03)	Leukotriene receptor antagonists (Montelukast)	42 (9.3)	27 (6.0)	12 (2.7)	6 (1.3)	34 (7.5)
R03DX	Other systemic drugs for obstructive airway diseases	11 (2.4)	*	*	*	*
R03DX05	*Omalizumab*	*	*	*	*	*
R03DX09	*Mepolizumab*	*	*	*	*	*
**H02AB, A07EC, P01BA02, L03, L04AD, AX, AA, J06BA**	**Drugs to treat auto-immune diseases**	1'057 (233.6)	628 (138.8)	417 (92.2)	556 (122.9)	1'213(268.1)
H02AB	Systemic glucocorticoids	*850 (187.8)*	*451 (99.7)*	*284 (62.8)*	*408 (90.2)*	*1'003 (221.7)*
L04AD, AX, AA, A07EC, P01BA02, L01XC02	Traditional immunosuppressants	463 (102.3)	453 (100.1)	429 (94.8)	468 (103.4)	591 (130.6)
A07EC02	*Mesalazine*	*66 (14.6)*	*65 (14.4)*	*67 (14.8)*	*75 (16.6)*	*106 (23.4)*
A07EC01	*Sulfasalazine*	*23 (5.1)*	*22 (4.9)*	*18 (4.0)*	*16 (3.5)*	*24 (5.3)*
P01BA02	*Hydroxychloroquine*	*44 (9.7)*	*42 (9.3)*	*38 (8.4)*	*41 (9.1)*	*52 (11.5)*
L04AX0	*Azathioprine*	*47 (10.4)*	*42 (9.3)*	*43 (9.5)*	*39 (8.6)*	*50 (11)*
L04AD02	*Tacrolimus*	*	*	*	*	*
L04AD01	*Ciclosporin*	*	*	*	*	*
L04AX03	*Methotrexate*	*	*	*	*	*
L01BA01	*Methotrexate*	*	*	*	*	*
L04AA06	*Myophenolic acid*	*	*	*	*	*
L04AA29	*Baricitinib*	*	*	*	*	*

L04AB, AC	Biologic DMARDS	92 (20.3)	81 (17.9)	53 (11.7)	37 (8.2)	86 (19.0)
L04AB	TNF alpha inhibitors	85 (18.8)	78 (17.2)	52 (11.5)	37 (8.2)	83 (18.3)
L04AB05	*Certolizumab*	*18 (4.0)*	*20 (4.4)*	*19 (4.2)*	*18 (4.0)*	*28 (6.2)*
L04AB02	*Infliximab*	*24 (5.3)*	*22 (4.9)*	*19 (4.2)*	*11 (2.4)*	*22 (4.9)*
L04AB01	*Etanercept*	*20 (4.4)*	*17 (3.8)*	*7 (1.5)*	*3 (0.7)*	*19 (4.2)*
L04AB04	*Adalimumab*	*17 (3.8)*	*15 (3.3)*	*	*	*17 (3.8)*
L04AB06	*Golimumab*	*	*	*	*	*
L04AC	Interleukin inhibitors	*	*	*	*	*
L04AC07	*Tocilizumab*	*	*	*	*	*
L04AC05	*Ustekinumab*	*	*	*	*	*
L04AA33	*Vedolizumab*	*	*	*	*	*
L01XC	Monoclonal antibodies	*	*	*	*	*
L01XC02	*Rituximab*	*	*	*	*	*
L01XC03	*Trastuzumab*	*	*	*	*	*
L01XC07	*Bevacizumab*	*	*	*	*	*
J06BA	Immunoglobulins, normal human	*	*	*	11 (2.4)	13 (2.9)
J06BA02	*Immunoglobulins, normal for intravascular administration*	*	*	*	*11 (2.4)*	*13 (2.9)*
	Multiple sclerosis specific drugs	63 (13.9)	38 (8.4)	5 (1.1)	8 (1.8)	35 (7.7)
L04AA23	*Natalizumab*	*21 (4.6)*	*14 (3.1)*	*	*	*14 (3.1)*
L03AX13	*Glatiramer acetate*	*22 (4.9)*	*12 (2.7)*	*	*	*13 (2.9)*
L03AB07	*Interferon beta-1 a*	*20 (4.4)*	*11(2.4)*	*	*	*12 (2.7)*
L04AX07	*Dimethylfumarate*	*	*	*	*	*
L04AA27	*Fingolimod*	*	*	*	*	*
**J05AR + J05AE01 / 03 /08 / 10 + J05AF05 / 07 / 11 +**	**HIV antiretrovirals**	60 (13.3)	62 (13.7)	79 (17.5)	87 (19.2)	98 (21.7)

**J05AG01 / 04 / 05 +**						
J05AR03	*Tenofovir disoproxil and emtricitabine*	*28 (6.2)*	*26 (5.7)*	*32 (7.1)*	*36 (8.0)*	*40 (8.8)*
J05AE03	*Ritonavir*	*16 (3.5)*	*15 (3.3)*	*25 (5.5)*	*27 (0.2)*	*29 6.4)*

* Indicates numbers below 10 and are not shown due to confidentiality reasons.

Table S3. Exposure to known or potential teratogens drugs during risk period and associated potential risks (weighted numbers).

Known or potential teratogenic drugs (ATC code)	Warnings regarding use during critical period	Risk period	Pregnancies exposed during risk period during study period (N)	Pregnancies exposed during risk period during study period (/10'000)
Methotrexate (L01BA01, L04AX03)	Risk of abortion. Malformations including face, skull, central nervous system, limbs, heart.	T1 (maximal risk between 8-10 GW)	*	*
Mycophenolic acid (L04AA06)	Risk of abortion. Malformations including cleft palate, ear deformities in up to 25% of exposed fetuses.	T1	*	*
Cyclophosphamide (L01AA01)	Malformations including the face, skull, eyes and central nervous system. Hematologic anomalies (anemia, leucopenia) in the newborn.	T1,T2 and T3	*,*,*	*,*,*
Carbimazole (H03BB01)	Malformations including aplasia cutis, choanal atreasia, tracheo-esophageal fistula in up to 4% of exposed fetuses.	T1 (maximal risk between 6-10 GW)	22	4.9
Lithium (N05AN01)	Increased risk of heart malformation, especially the Ebstein malformation in 2.5% of exposed babies (including 1% of Ebstein disease vs 0.005%).	T1 (until GW 10)	*	*
Dolutegravir (J05AR13)	Suspicion of an increased risk of neural tube defects.	T1 (Maximal risk between 0-4 GW)	*	*

* Indicates numbers below 10 and are not shown due to confidentiality reasons.

Table S4. Exposure to known fetotoxic drugs during risk period and associated potential risks (weighted numbers).

Known or potential fetotoxic drugs (ATC code)	Warnings regarding use during critical period	Risk period	Pregnancies exposed during risk period during study period (N)	Pregnancies exposed during risk period during study period /10'000 95%CI)
Agents acting on the RAA system. (C09)	Renal toxicity which can induce oligo/anamnios, with potential pulmonary, skull hypoplasia and reduction of the extremities. Neonatal renal insufficiency.	T2 and T3	12, *	2.7, *
Psychotropic drugs - Antidepressants ; - Antipsychotics ; - Benzodiazepines and related drugs ((N06A, N05A, N05AN, N05BA, N05CD, N05CF)	-1/3 newborns present transient neonatal symptoms (serotoninergic toxicity or weaning symptoms) ; - Risk of transient extra-pyramidal symptoms in the newborn ; - Prenatal exposure to benzodiazepines with long half-life in may cause impregnation symptoms (sleepiness, succion problems, hypotension, respiratory distress), whereas short half-life substances may cause weaning symptoms (agitation, hyperexcitability)	T3	771	170.4
Trastuzumab (L01XC03)	Oligo-anhydramnios	T2 and T3	0, 0	0.0
Rituximab (L01XC02)	Neonatal B cell depletion	T2 and T3	0, 0	0.0

* Indicates numbers below 10 and are not shown due to confidentiality reasons.

Table S5. Exposure to potential teratogenic drugs (risk found in animal studies but not in human data) during risk period and associated potential risks (weighted numbers).

Potential teratogenic drugs (ATC code)	Warnings regarding use during critical period	Risk period	Pregnancies exposed during risk period during study period (N)	Pregnancies exposed during risk period during study period (%, 95%CI)
Propylthiouracil (H03BA02)		T1	*33*	7.3
Mitoxantrone (L01DB07)	Contraception is recommended 4 months after the last administration	Pre-pregnancy and T1.	0, 0	0.0, 0.0
Fingolimod (L04AA27)	Contraception is recommended 2 months after the last administration		*, *	*,*
Teriflunomide (L04AA31)			0, 0	0.0, 0.0
Leflunomide (L04AA13)			0, 0	0.0, 0.0
Dimethylfumarate (L04AX07)	Contraception is recommended 3.5 months after the last administration.		*, *	*,*
Cladribine (L01BB04, L04AA40)			0, 0	0.0
Alemtuzumab (L04AA34)	Contraception is recommended 4 months after the last administration.		0, 0	0.0
Tofacitinib (L04AA29) [90]			*,*	*,*
Baricitinib (L04AA37) [91]			0, 0	0.0

* Indicates numbers below 10 and are not shown due to confidentiality reasons.
